# *Caenorhabditis* Intervention Testing Program: the herbicide diuron does not robustly extend lifespan in nematodes

**DOI:** 10.17912/micropub.biology.000448

**Published:** 2021-09-23

**Authors:** Hadley C Osman, Christine A Sedore, E Grace Jackson, Elena T Battistoni, David Hall, Anna Foulger, Mark Lucanic, Max Guo, Monica Driscoll, Patrick Phillips, Gordon J Lithgow

**Affiliations:** 1 The Buck Institute for Research on Aging, Novato, California 94945, USA; 2 Institute of Ecology and Evolution, University of Oregon, Eugene, Oregon 97403, USA; 3 Division of Aging Biology, National Institute on Aging, Bethesda, Maryland 20892, USA; 4 Department of Molecular Biology and Biochemistry, Rutgers University, Piscataway, New Jersey 08854, USA

**Figure 1:**
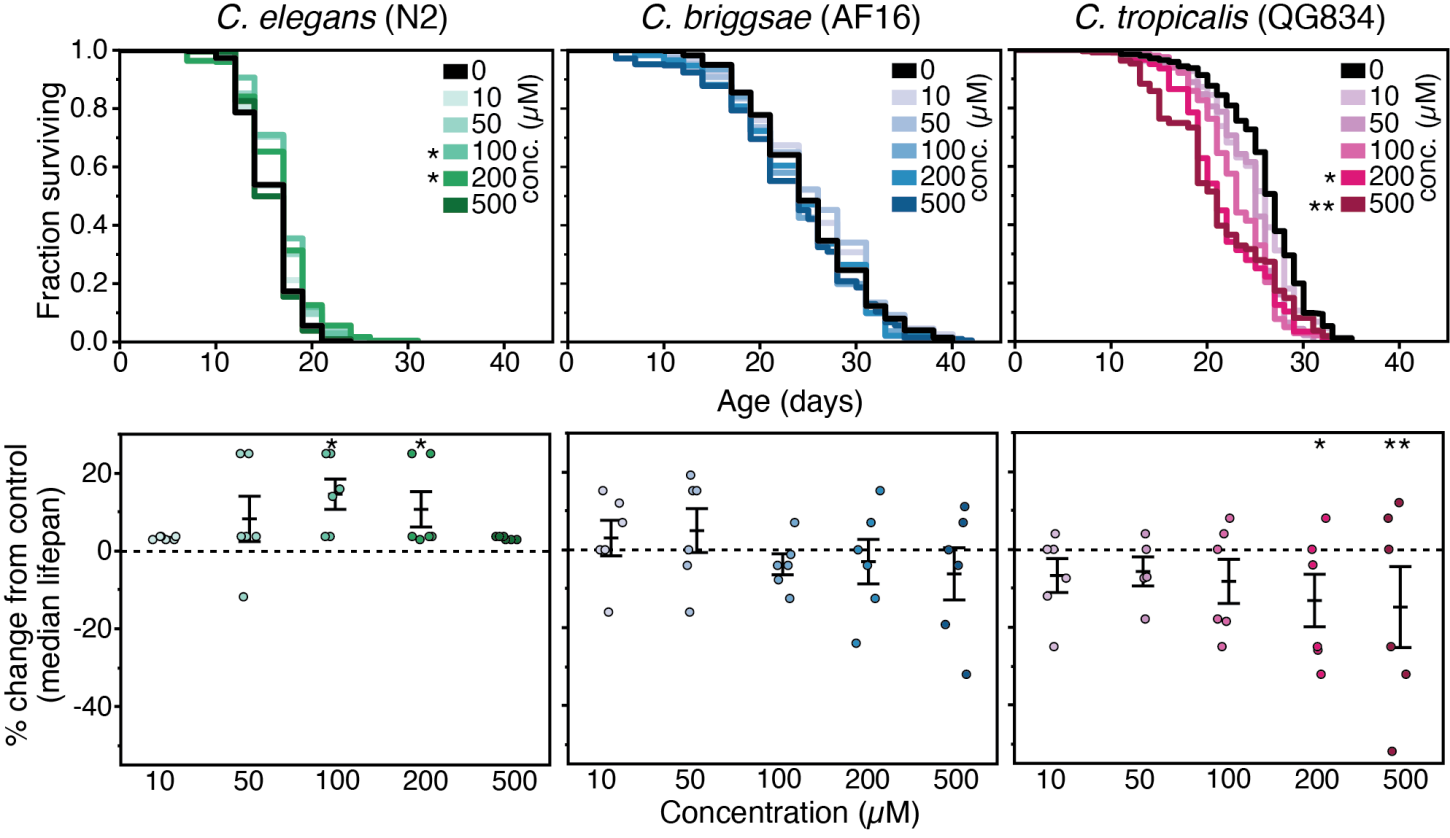
Lifespan results for three *Caenorhabditis* strains on various concentrations of diuron. Mean lifespan differed significantly in *C.* *elegans* N2at 100 µM (8.7% increase, *p*=0.0145) and 200 µM (6.2% increase, *p*=0.0199) and *C.* *tropicalis* QG834at 200 µM (16.5% decrease, *p*=0.01628), and 500 µm (18.3%decrease, *p*=0.00335). The Kaplan-Meier lifespan curves represent pooled replicates across two trials. The dot plots show the percent change in median lifespan with each data point representing an individual plate replicate as compared to its control average for a given trial. Bars represent the mean +/- the standard error of the mean. Asterisks represent *p*-values from the Cox Proportional Hazard model (Cox proportional hazard mixed-model using the coxme v.2.2-5 package in R (Therneau 2012)) such that **p<0.01 and *p<0.05.

## Description

The *Caenorhabditis* Intervention Testing Program (CITP) is a multi-institutional, National Institute on Aging (NIA)-funded consortium with the goal of identifying compounds that will robustly extend lifespan with reproducible effects across genetically diverse *Caenorhabditis* species and strains (Lucanic **et al.*,* 2017). Compounds are prioritized for testing based on computational prediction for lifespan or healthspan effects (Coleman-Hulbert **et al.*,* 2019), predicted or known interactions with documented lifespan-regulating pathways, or previous reports for lifespan or healthspan extension in laboratory animals. In this case, diuron was of interest to CITP based on the report of a positive effect on the lifespan of C. elegans strain TJ1060 (*spe-9;fer-15*) (Lucanic *et al.*, 2018; personal communication). Diuron (1-(3,4-dichlorophenyl)-3,3-dimethylurea) is an herbicide that inhibits photosynthesis by binding photosystem II (Haynes **et al.*,* 2000). Diuron has been previously tested in nematode species for toxicity (Neury-Ormanni **et al.*,* 2019) and reproductive effects (Mugova **et al.*,* 2018), and in both cases showed detrimental effects at high doses only.

We assayed lifespan in response to diuron exposure at five different concentrations across three species of *Caenorhabditis* nematodes following our previously published protocols (Lucanic **et al.*,* 2017). Briefly, animals were age synchronized by performing a four-hour long egg lay on Nematode Growth Media (NGM) agar plates seeded with *E. coli* OP50-1. An average of 40 day one adult worms were transferred in triplicate to 30 mm NGM agar plates seeded with the *E. coli* strain OP50-1 and containing 50 µM 5-Fluoro-2′-deoxyuridine (FUdR). Diuron (Sigma Aldrich) was dissolved in DMSO at a concentration such that 7.5 µl of diuron solution and 125 µl of water were added to each 3 mL plate to obtain final concentrations of 10 µM, 50 µM, 100 µM, 200 µM, and 500 µM. Control plates were treated with 125 µl water and 7.5 µl DMSO. Nematodes were maintained at 20˚C and transferred to fresh plates on days 1, 2, and 4 (*C*. *tropicalis*) or 5 (*C.* *briggsae* and *C.* *elegans*) of adulthood, and once per week thereafter. Animals were scored manually for movement, and those that showed no movement, either spontaneous or after gentle perturbation with a 0.2 mm diameter platinum wire, were scored as dead. Animals that had burrowed or escaped the NGM plate were censored in the final analysis. The experiment was repeated in two biological replicates, with an average of 40 animals per condition across three technical replicates, for a total of 4,920 animals.

Our results indicate that diuron does not exert robust positive effects on lifespan across three species of *Caenorhabditis* nematodes (Fig. 1). *C. elegans* strain N2 showed a weakly significant, non-robust increase in mean lifespan at 100 µM and 200 µM. Conversely, *C. tropicalis* showed a significant, robust, and dose dependent decrease in lifespan at 200 µM and 500 µM, with high variability observed across biological replicates. *C. Briggsae* strain AF16 showed no significant increase or decrease in lifespan. Diuron was of interest to CITP due to effects on lifespan in a preliminary screen on *C. elegans* strain TJ1060 (Lucanic **et al.*,* 2018; personal communication), results that were not reproduced when tested under CITP test conditions and in CITP strains. Diuron has been shown to have toxic effects on some strains of nematodes in high concentrations (Neury‐Ormanni **et al.*,* 2019), consistent with our results in *C. tropicalis* (Fig. 1). Under CITP testing, the lifespan extension effect observed was small and restricted to the *elegans* species of *Caenorhabditis*. For similar initial trial lifespans in the CITP, a lifespan increase of 10% or higher is an indication that an intervention merits further testing. For example, NP-1 showed a consistent lifespan increase of ~30%, and therefore this compound was subject to extended testing by three CITP labs (Lucanic *et al.*, 2017). Diuron did not exceed an increase of 8.7%, conferred variable effects, and thus did not merit further testing in the CITP platform.

## Reagents

**Table:** 

Reference strain	Species	Available from
N2	*Caenorhabditis elegans*	CGC
QG834	*Caenorhabditis tropicalis*	CGC
AF16	*Caenorhabditis briggsae*	CGC
OP50-1	*Escherichia coli*	
